# Nutritional skewing of conceptus sex in sheep: effects of a maternal diet enriched in rumen-protected polyunsaturated fatty acids (PUFA)

**DOI:** 10.1186/1477-7827-6-21

**Published:** 2008-06-09

**Authors:** Mark P Green, Lee D Spate, Tina E Parks, Koji Kimura, Clifton N Murphy, Jim E Williams, Monty S Kerley, Jonathan A Green, Duane H Keisler, R Michael Roberts

**Affiliations:** 1 Division of Animal Sciences, University of Missouri, Columbia MO 65211, USA; 2 AgResearch Ltd., Ruakura Research Centre, Hamilton 3240, New Zealand; 3 National Institute of Livestock and Grassland Science, Reproductive Physiology Lab., Tochigi 329-2793, Japan; 4 Christopher S. Bond Life Sciences Center, University of Missouri, Columbia MO 65211, USA

## Abstract

**Background:**

Evolutionary theory suggests that in polygynous mammalian species females in better body condition should produce more sons than daughters. Few controlled studies have however tested this hypothesis and controversy exists as to whether body condition score or maternal diet is in fact the determining factor of offspring sex. Here, we examined whether maternal diet, specifically increased n-6 polyunsaturated fatty acid (PUFA) intake, of ewes with a constant body condition score around the time of conception influenced sex ratio.

**Methods:**

Ewes (n = 44) maintained in similar body condition throughout the study were assigned either a control (C) diet or one (F) enriched in rumen-protected PUFA, but otherwise essentially equivalent, from four weeks prior to breeding until d13 post-estrus. On d13, conceptuses were recovered, measured, cultured to assess their capacity for interferon-tau (IFNT) production and their sex determined. The experiment was repeated with all ewes being fed the F diet to remove any effects of parity order on sex ratio. Maternal body condition score (BCS), plasma hormone and metabolite concentrations were also assessed throughout the study and related to diet.

**Results:**

In total 129 conceptuses were recovered. Ewes on the F diet produced significantly more male than female conceptuses (proportion male = 0.69; deviation from expected ratio of 0.5, P < 0.001). Conceptus IFNT production was unaffected by diet (P > 0.1), but positively correlated with maternal body condition score (P < 0.05), and was higher (P < 0.05) in female than male conceptuses after 4 h culture. Maternal plasma hormone and metabolite concentrations, especially progesterone and fatty acid, were also modulated by diet.

**Conclusion:**

These results provide evidence that maternal diet, in the form of increased amounts of rumen-protected PUFA fed around conception, rather than maternal body condition, can skew the sex ratio towards males. These observations may have implications to the livestock industry and animal management policies when offspring of one sex may be preferred over the other.

## Background

A number of studies have demonstrated a significant variation from the expected 1:1 birth sex ratio in many mammalian species [1-5]. Perhaps the most studied factors considered to influence sex ratio are maternal diet and body condition. Based on evolutionary theory, mothers should adjust their birth sex ratios in relation to the future reproductive benefits that would accrue to them in terms of their ability to pass on their genes to future generations [1-5]. In polygynous species, in particular where a small proportion of males, usually those that are larger, stronger and more aggressive are most reproductively successful, females in good condition should bias their offspring in favor of males, since that sex would yield the highest return of fitness. The reverse would be true for females in poor body condition; they would be anticipated to invest in daughters rather than sons [1]. Although this theory is attractive, observations have often been contradictory, leading some to dismiss the relevance of the hypothesis [2-5], particularly as no proven mechanism for adjusting sex ratio has emerged. Several hypothetical mechanisms to explain sex ratio adjustment focus on differences in maternal physiology around conception, implantation or in relationship to early development [2,6-10].

The sex-allocation hypothesis of Trivers and Willard [1-5] has been applied successfully to ruminant species, although meta-analysis of 37 studies only identified a weak positive correlation between maternal condition and sex ratio [5]. Particular confusion relates to the term 'maternal body condition', which is based upon morphological assessment and often made without knowledge of actual body fat deposition, food quality and/or intake. Measures of maternal condition taken prior to conception provide stronger evidence of a relationship with sex ratio, than those that rely on morphological or physiological measures of condition taken post-conception [1-5].

There have been surprisingly few controlled experiments to determine whether maternal nutrition influences the sex ratio of offspring [4]. Studies can be separated into those focusing upon dietary restriction and its timing versus those concerned with dietary composition. In golden hamsters [11] and house mice [12] diet restriction during pregnancy leads to a reduction in the number of males born. In contrast, alteration of the dietary composition, specifically the content of oils and fat that increase the caloric intake before pregnancy, can skew the offspring sex towards males in opossum [13] and mice [14,15]. With the exception of one report on fallow deer [16], where females on a higher calorie intake produced more sons than daughters, to the authors' knowledge, there have been no experimental studies performed in livestock that have examined whether nutrition of the mother can affect sex ratio under controlled experimental conditions. Two large retrospective studies of lactating dairy cows have however found indirect measures of better nutrition to be positively associated with a skewing of sex ratio towards males [17,18]. Clearly, controlled studies performed to determine how maternal diet influences sex of offspring would be beneficial in livestock industries in which pregnancy outcome strongly influences the efficiency of producing milk or meat [19].

Ruminants are problematic when it comes to studying the effects of fat and oil supplementation on parameters such as reproductive performance [20]. First, the fatty acid content of fats varies widely. Typically, unprocessed plant oils are rich in long chain polyunsaturated fatty acids (PUFA), while fats derived from animals contain mainly saturated fats and a variable proportion of the C_18:1 _monounsaturated fatty acid, oleic acid. Second, the majority of the fatty acids consumed are utilized by rumen flora. Only in monogastric species of mammals does blood composition reflect the fatty acid content of the diet [21]. In ruminants, 70 to 90% of PUFA supplied become hydrogenated before they reach the small intestine and are utilized [22-24]. However, some sources of plant oils are more resistant to the rumen flora than others [22], resulting in >25% PUFA uptake. Recently, dietary supplements containing essential fatty acids that are protected from lipolysis and bio-hydrogenation in the rumen have become commercially available, thereby allowing the effects of enhanced PUFA uptake to be examined.

Evidence exists that alteration of the concentration and ratio of n-6 and n-3 PUFAs in feed can influence several reproductive parameters in ruminants, specifically n-6 PUFAs, which can be converted to longer chain gamma linolenic and arachidonic acid, precursors of prostaglandins [21,22]. The manipulation of prostaglandin synthesis and metabolism can be profound, with affects on follicle development, ovulation, corpus luteum (CL) function and hormone secretion being reported [21]. Therefore, the hypothesis underpinning the present experiment was that the fat composition of the diet, specifically the n-6 PUFA concentration, fed to ewes around the time of conception would influence the sex ratio of offspring born.

## Methods

### Animals and diets

Non-parous Romanov crossbred ewes (n = 44) not more than 30 months of age were divided into two weight matched groups and assigned either a control (C) or a diet rich in n-6 polyunsaturated fats (F) (Table 1). The C diet was characteristic of the type of ration used in the sheep industry for dry-lot managed ewes. The F diet differed primarily only from the C diet in that it was supplemented with fat. To ensure its effectiveness the fat component, Neofat (Morgan Co.; Paris, IL), a hydrolyzed saponified derivative of soybean oil, was used to "protect" the fatty acids from the rumen microflora and allow digestion and absorption in the intestines. The diets formulated were approximately isocaloric and isonitrogenous, as well as isoenergetic in terms of energy distributed between the different nutrient sources. Analysis of diets confirmed their composition (Livestock Nutrition Laboratory Services, Columbia, MO). Two weeks prior to starting the experiment, both groups of ewes were managed in dry-lots and offered twice daily the C diet to meet basal maintenance requirements (0.8 kg/ewe/day). Subsequently, ewes were housed on average in pens of three. One group was fed the C diet (Group A) and the other the F diet (Group B) for 4 weeks prior to breeding and until day 13 post-estrus. Estrous cycles were synchronized via intramuscular administration of 30 mg, (two injections of 15 mg; 4 h apart) of the prostaglandin F_2α _analogue, Lutalyse (Pharmacia & Upjohn Company, Kalamazoo, MI) nine days apart. Estrus (d 0) was identified by the use of a marker-harnessed vasectomized ram. Thereafter, ewes were checked twice daily for estrus and bred at the following natural estrus to at least two rams, each three times successfully, twice a day until estrus concluded. Rams were not fed either of the diets. On day 13 post-estrus each uterine horn was surgically flushed with 37°C sterile modified phosphate buffered saline (m-PBS; 30 ml) containing 5.56 mM glucose to recover conceptuses as described in previous studies from this laboratory [25,26]. The developmental stage (spherical or elongated), length and width, as well as status (intact or fragmented) of each conceptus was recorded prior to being individually cultured. The number of CL was also recorded to determine whether the value matched the number of conceptuses recovered in the flush. Following surgery, ewes were allowed to recover for six weeks while housed in dry-lots and fed the C diet. In *Phase 2*, ewes in both Group A and B were fed the F diet to account for the possibility of parity order on sex ratio and to increase the sample size, since feeding of the C diet in *Phase 1 *was undertaken to demonstrate that, as identified by the literature, feeding a standard diet did not skew sex ratio. An identical schedule to *Phase 1 *was followed, although on day 13 post-estrus ewes were euthanized rather than being subjected to a second surgery. Reproductive tracts were examined for the number of CL present on the ovaries and flushed to recover conceptuses.

**Table 1:** Ingredient and nutrient compositions of the diets (Control and Fat) fed to dry-lot managed ewes

	**Diet**	
	
**Ingredient Composition (% of diet dry matter)**	**Control (C)**	**Fat (F)**
Cracked Corn	36.7	27.5
Soybean Meal	9.0	11.0
Dehydrated Alfalfa	5.2	5.2
Cottonseed hulls	43.6	50.9
Molasses	4.8	0.0
Neofat	0.0	4.6
Minerals	0.6	0.7
Vitamins	0.1	0.1

**Total**	**100**	**100**

	**Diet**	
	
**Nutrient Composition**	**Control (C)**	**Fat (F)**

Dry Matter (%)	88.2	91.6
Crude Fiber (% of dry matter)	31.9	29.2
Crude Protein (% of dry matter)	11.0	11.5
Crude Fat (% of dry matter)	1.9	4.8
Metabolizable Energy (Mcal/kg)	2.5	2.4

Individual ewe weights and body condition scores (BCS) were assessed weekly. BCS was assessed following industry standard guides by 3 experienced assessors, specifically by palpation of the thoracic and vertebral regions of the spinal column (loin and rump), the ribs, the tuber sacrale (hip bones), the tuber ischii (pin bones), the tail head and the thigh region. BCS were calculated as the mean score of three assessors throughout the study (August to January), and dietary intake adjusted on an individual basis to maintain weights and BCS between 3.0 to 3.5 (Scale: 1 = emaciated to 5 = severely obese). Weekly blood samples were collected via jugular venipuncture to determine the concentration of metabolic parameters (glucose, insulin, IGF-I, leptin, and non-esterified fatty acids (NEFAs)). Daily samples were also collected during pregnancy (d0–d13) to ascertain progesterone concentrations. Plasma was harvested from blood by centrifugation for 15 min at 1500 × *g *and stored at -20°C for subsequent hormone analysis. The study was completed in accordance with University of Missouri Animal Care and Use Committee Protocol 3716.

### Conceptus culture and IFNT measurement via antiviral assay

IFNT is generally regarded as the major signal for maternal recognition of pregnancy in ruminant ungulates [27-29] and its production is a useful indication of conceptus well being. Accordingly, we measured the amount of IFNT (assessed by its antiviral activity) released by conceptuses into the medium during their culture *in vitro *subsequent to their recovery from the uterus.

Conceptuses were thoroughly washed in m-PBS prior to individual culture in 500 μl of Pig Minimum Essential Medium (Pig MEM) [30], consisting of MEM (Gibco Lifesciences; Rockville, MD) supplemented with 10% FBS (Harlan BioProducts; Indianapolis, IN), 1% 100 × Non essential amino acids (Sigma Chemical Co.; St. Louis, MO), 16.68 mM D-Glucose (Sigma Chemical Co.; St. Louis, MO) and 0.2 IU Insulin (Sigma Chemical Co.; St. Louis, MO). Conceptuses were cultured for 24 h under 5% O_2 _5% CO_2 _90% N_2 _at 39°C. Medium was collected at 4 h, replaced, and at the end of culture (24 h) again collected to measure IFNT concentration. At 24 h a small portion of each conceptus was removed for sex determination via PCR.

Medium collected after 4 and 24 h culture was analyzed for antiviral activity content via a cytopathic reduction assay (n = 18) involving Madin-Darby Bovine Kidney (MDBK) cells challenged with a vesicular stomatitis virus [31]. The standard used was recombinant boIFNT1A of known antiviral activity (7.5 × 10^7 ^IU mg^-1^) [32] that had been standardized against human IFNA (PBL Biomedical Laboratories; Piscataway, NJ). The assay protocol used has previously been described in detail [33].

### Sexing of conceptuses

Conceptus DNA was extracted in 5 μl of lysis buffer (20 mM Tris-HCl, 0.9% Tween-20, 0.9% NP-40), pH 7.5 with Proteinase K (final concentration of 0.4 mg/ml) at 55°C for 30 mins followed by 10 mins at 98°C. Sexing was performed by PCR amplification of a Y-specific DNA sequence [34]. As a control reaction, a non-sex specific ovine autosomal sequence [35] was co-amplified to demonstrate the presence of conceptus DNA. Ovine male and female genomic DNA (20 pg), as well as water samples were included as controls and treated exactly the same as conceptus DNA. The PCR amplification (93°C for 30 sec followed by 30 cycles of 93°C for 1 min, 61°C for 1 min and 68°C for 1 min) was set up essentially as described by Larson et al. [33], except with sheep autosomal primers at 2 pmol and Y-specific primers at 5 pmol. After amplification, PCR products were visualized by electrophoresis on a 4% agarose gel. The autosomal primers yielded a band at 256 bp and the Y-specific primers a band at 301 bp.

### Plasma assays

Progesterone and insulin concentrations were determined via the use of a Coat-A-Count kit (DPC; Los Angeles, CA) following the manufacturer's instructions. The assay sensitivity for progesterone (n = 6) and insulin (n = 3) was 0.2 ng ml^-1 ^and 4.6 μIU ml^-1 ^with a specific binding of 55.2% and 34.4% respectively. The intra- and inter-assay coefficients of variation were 3.6% and 5.9% for progesterone, 4.2% and 6.4% for insulin. Glucose concentrations were determined by using a commercially available colormetric assay kit (ThermoDMA; Louisville, CO) as described in the manufacturer's instructions. Assay sensitivity (n = 44) was 0.04 mmol L^-1 ^with intra- and inter-assay coefficients of variation of 6.3% and 12.4% respectively. NEFAs were measured using the Wako NEFA C colormetric assay kit (Wako Chemicals USA; Richmond, VA) as described [36]. Assay sensitivity (n = 31) was 0.01 mEq L^-1 ^with intra- and inter-assay coefficients of variation of 4.1% and 9.8% respectively. Plasma leptin concentrations were determined via a double antibody radioimmunoassay as previously described [37]. Assay sensitivity (n = 6) was 0.8 ng ml^-1 ^and the specific binding 38.6%. The intra- and inter-assay coefficients of variation were 7.6% and 11.7% respectively. Plasma IGF-I concentrations were measured after acidified extraction via a double antibody radioimmunoassay [38]. Assay sensitivity (n = 3) was 8.6 ng ml^-1 ^and the specific binding 41.8%. The intra- and inter-assay coefficients of variation were 10.3% and 13.5% respectively.

### Statistical analysis

Maternal body weight, BCS and weekly plasma hormone concentrations were calculated for two time periods. Firstly, a mean value was determined that included the whole experimental time period, 4 weeks prior to breeding (start of diet) to uterine flushing (d13 post-estrus). A second mean value was calculated from data collected from the time of breeding to uterine flushing (d0 to d13). Daily progesterone concentrations for each ewe were analyzed as area under the curve (AUC) d2 to d13 post-estrus and AUC d2 to d5 post-estrus. In addition, linear regression analysis of progesterone concentrations between d2 and d5 allowed us to calculate the slope of the curve for the increase in progesterone that occurs as the CL first become functional. This variable was also included in the statistical model. As with other studies investigating sex ratio, the sex ratio (proportion male) was compared with an expected 1:1 ratio by a corrected γ^2 ^procedure as well as by using Binominal analysis. These analyses were performed for both the C and F diets. Sex ratio differences between ewes on the C and F diets were assessed for *Phase 1*. All analyses were run by using the Proc Mix Glimmix procedure of SAS software version 9.1 (SAS institute, Cavy, NC). A general linear mixed model with appropriate link functions for data with a Poissonian distribution was used to investigate the effect of diet on the proportion of male conceptuses and on plasma hormone concentrations. The Phase of the experiment (*Phase 1 or Phase 2*) was treated as a repeated measure. Average weight, weight change, average BCS, change in BCS, pregnancy status, conceptus and corpora lutea (CL) number were included in the model and all interactions between the main effects were analyzed.

Conceptuses were classified as either intact or not after recovery. The majority of conceptuses identified as non-intact had only minimal damage to the trophectoderm. However, since there was some tissue loss from damaged embryos only data from conceptuses deemed to be intact at recovery were included in the analysis, since their true dimensions were unknown and IFN production could potentially have been affected. IFNT antiviral values were log-transformed, as the distribution of the raw data was skewed. A linear mixed model was employed with hour (4 or 24 h) and Phase as repeated measures. The length, width, calculated area ([length × 2] width) and sex of the conceptus were included as covariates. Phase, maternal diet, progesterone concentration, average weight, weight change, average BCS and BCS change were also included in the model. Results are presented as the least squared mean ± SEM unless otherwise stated.

## Results

### Weight, BCS, pregnancy rates and embryo recovery

BCS did not differ (P > 0.1) between ewes on the C and F diets (Table 2). Nor was there any significant change in BCS scores in either group over the course of the experiment. The mean weight of the ewes was also the same (P > 0.1) in the two dietary groups, and ewe weights remained relatively constant over the five months of the study (see Table 2).

**Table 2:** Mean physical, metabolic and hormonal measures of ewes shown by diet (Control or Fat)

	**Diet Fed to Ewes**	
	
**Physical, Metabolic or Hormonal Ewe Measures**	**Control (Diet C)**	**Fat (Diet F)**
Mean weight (kg)	54.7 ± 1.0	56.9 ± 1.4
Change in weight (kg)	0.9 ± 1.1	1.6 ± 0.8
Mean BCS	3.2 ± 0.1	3.3 ± 0.1
Change in BCS	0.1 ± 0.05	0.1 ± 0.1
Average plasma glucose (mmol L^-1^)	3.40 ± 0.1	3.91 ± 0.2
Breeding plasma glucose (mmol L^-1^)	3.32 ± 0.2	3.64 ± 0.2
Average plasma leptin (ng ml^-1^)	14.0 ± 1.0	13.7 ± 0.9
Breeding plasma leptin (ng ml^-1^)	14.5 ± 1.2	14.1 ± 1.2
Average plasma IGF-I (ng ml^-1^)	148.2 ± 9.4	141.4 ± 8.4
Breeding plasma IGF-I (ng ml^-1^)	140.4 ± 8.4	136.5 ± 7.4
Average plasma insulin (μIU ml^-1^)	74.1 ± 6.3	63.1 ± 7.4
Breeding plasma insulin (μIU ml^-1^)	76.8 ± 7.9	64.8 ± 7.7
Average plasma NEFA (mEq L^-1^)	0.21 ± 0.01	0.24 ± 0.03*
Breeding plasma NEFA (mEq L^-1^)	0.170 ± 0.02	0.181 ± 0.02
Plasma progesterone (ng ml^-1^) AUC d2 to d5^†^	5.5 ± 0.3	4.6 ± 0.3 *
Plasma progesterone (ng ml^-1^) AUC d2 to d13^†^	56.1 ± 2.7	50.0 ± 2.0*
Plasma progesterone (ng ml ^-1 ^day ^-1^) Slope d2 to d5^†^	1.3 ± 0.1	1.6 ± 0.1**

AUC = Area under the curve. ^†^Data from *Phase 1 *only. Superscripts indicate values are significantly different within rows * (P < 0.05), ** (P < 0.01).

Ewes were bred twice, in October 2003 and January 2004. Fertility and breeding rates of the ewes were high in each of the two phases of the study. In *Phase 1*, 43 of the 44 ewes (98%) demonstrated estrus and were bred successfully. On d13 post-breeding, 33 ewes (77%) had at least one conceptus in their uterine flushings. In total, 43 intact (including 10 spherical) and 12 largely intact (slightly damaged trophectoderm) conceptuses were recovered. In *Phase 2*, one ewe was euthanized after complications post-surgery and, of the remaining 43 ewes, 41 (95%) were bred successfully. One ewe did not demonstrate estrus throughout the whole study and was excluded from all analyses. Of the 41 ewes bred in *Phase 2*, 37 (90%) were determined as pregnant (44 intact and 30 largely intact conceptuses). Tract abnormalities from the first surgery were evident in two of the four non-pregnant ewes. In total, 129 conceptuses (87 intact and 42 largely intact) were recovered in the two phases of the study. Overall the mean number of conceptuses per ewe (1.8 ± 0.1), as well as the mean number of CL (2.5 ± 0.1), were not different (P > 0.1) between *Phases *or diets. In addition, no differences with respect to these parameters were identified when only data from pregnant ewes were analyzed: mean number of conceptuses per ewe (1.8 ± 0.1; Diet C 1.6 ± 0.1, Diet F 1.8 ± 0.1) and CL (2.5 ± 0.1; Diet C 2.5 ± 0.1, Diet F 2.5 ± 0.1).

### Conceptus dimensions and sex

Intact conceptuses recovered in *Phase 2*, which was conducted later in the breeding season (January), were larger (P < 0.001) than those in *Phase 1*, which were recovered in October at the beginning of the season. Conceptus length (*Phase 1*, 3.9 ± 0.6 mm; *Phase 2*, 18.3 ± 3.0 mm), width (*Phase 1*, 0.9 ± 0.03 mm; *Phase 2*, 1.1 ± 0.04 mm) and area (*Phase 1*, 6.9 ± 1.1 mm^2^; *Phase 2*, 43.4 ± 8.8 mm^2^) all differed between *Phases*. Accordingly, we only used data from *Phase 1 *to determine whether there was an effect of maternal diet on the size of intact conceptuses. Maternal diet did not affect (P > 0.1) the length, width or the calculated surface area of the recovered conceptuses.

Conceptus sex was analyzed by PCR (Table 3). The complete data set (n = 129) included all intact conceptuses, plus those that were minimally damaged, and from ewes where the number of CL matched the number of whole and minimally damaged conceptuses recovered from a ewe. In ewes fed the F diet, the sex ratio (proportion male) was skewed towards males (69%; deviation from anticipated 0.5 value P < 0.001). Although the number of female conceptuses was numerically greater than the number of male conceptuses for ewes on the C diet, the fraction was not significantly different from the expected 0.5 value, i.e. the control diet did not cause sex ratio skewing.

**Table 3:** The sex of the total number and (intact number) of conceptuses recovered on day 13 post-estrus shown by individual phase and maternal diet (Control vs. Fat)

			**Ewes**		**Conceptus Sex**		
				
**Phase**	**Ewe group**	**Maternal diet**	**Bred**	**Pregnant**	**Female**	**Male**	**Male ratio**
1	A	Control	21	13 (12)	13 (11)	8 (8)	0.38
1	B	Fat	22	20 (17) ^†^	14 (9)	20 (15)	0.58
2	A	Fat	21	19 (14)	12 (9)	26 (14)	0.68*
2	B	Fat	20	18 (13)	7 (7)	29 (14)	0.81**

		**Control**	**21**	**13 (12)**	**13 (11)**	**8 (8)**	**0.38**
		
**Combined**		**Fat**	**63**	**57 (44)**	**33 (25)**	**75 (43)**	0.69**

The number of ewes in parentheses identifies the number from which intact conceptuses were recovered.

^†^Conceptuses recovered from one ewe were dropped at surgery and unable to be sexed. Male ratio with superscripts are different from the expected 0.50 ratio * (P < 0.05), ** (P < 0.001).

Differences in intact conceptus size between sexes were analyzed within phase. No differences in length (*Phase 1*, Females 4.07 ± 1.02 mm vs. Males 2.81 ± 0.53 mm; *Phase 2*, Females 22.81 ± 5.52 mm vs. Males 15.73 ± 3.89 mm) or width (*Phase 1*, Females 0.82 ± 0.05 mm vs. Males 0.88 ± 0.04 mm; *Phase 2*, Females 1.20 ± 0.09 mm vs. Males 1.05 ± 0.04 mm) measurements were identified.

### IFNT anti-viral activity of culture media (4 and 24 h)

**Figure 1:**
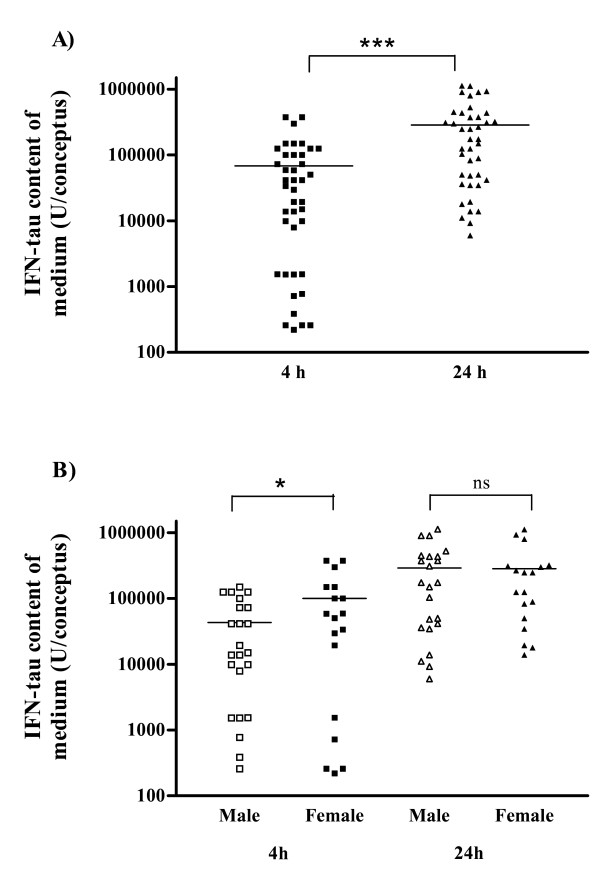
Scatter diagram showing the range in interferon-tau (IFNT) content of the medium (U per conceptus) between A) 4 and 24 h of culture (n = 41), B) between male (n = 23) and female (n = 18) intact conceptuses after 4 and 24 h culture. Mean values are indicated by horizontal lines. * P < 0.05, *** P < 0.001, ns = not significant (P > 0.1).

IFNT production by intact conceptuses was significantly less (P < 0.001) in *Phase 1 *(287,016 ± 50,275 U) than in *Phase 2 *(3,635,217 ± 1,431,133 U) of the study, an anticipated result since the conceptuses in *Phase 1 *were smaller. Not unexpectedly, the amount of IFNT activity recovered after 4 h of culture was less than that recovered in the subsequent 20 h of culture (*Phase 1*, 68,308.6 ± 14,727.1 U and 287,015.9 ± 50,274.3 U, respectively: Fig. 1.).

Maternal diet had no affect (P > 0.1) on IFNT production. Conceptus sex did, however, influence IFNT content of the medium, with female conceptuses producing more (P < 0.05) IFNT by 4 h than male conceptuses. It is unclear whether these differences could be attributed to the slightly larger (but statistically insignificant) size of the female conceptuses (see previous section) However, no differences in IFNT production was found between sexes at 24 h (Fig. 1).

**Figure 2:**
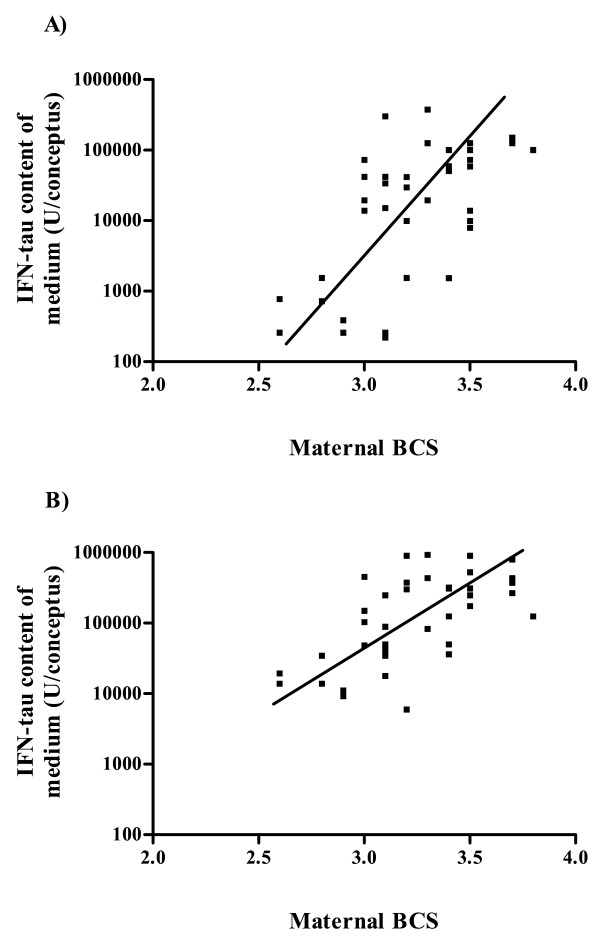
Linear regression analysis of average maternal body condition score (BCS; scale 1 = emaciated to 5 = obese) and interferon-tau (IFNT) content of the medium (U per conceptus) of intact conceptuses at A) 4 h (P < 0.01, r^2 ^= 0.12) and B) 24 h (P < 0.01, r^2 ^= 0.18) of culture (n = 41).

The production of IFNT was positively correlated with conceptus size (area and length; both P < 0.01), and, interestingly, with maternal BCS (P < 0.05, Fig. 2.).

### Metabolic and hormone concentrations in plasma of ewes

The effects of diet on the concentration of maternal circulating metabolites and hormones concentrations on the weekly blood samples from all ewes throughout the study (average concentration), as well as samples collected daily in the period from breeding to recovery on d13 (breeding concentration) are summarized (Table 2).

Diet did not significantly affect either average or breeding plasma glucose concentrations. There was however a slight increase in circulating glucose in the heavier ewes (P < 0.05) and in those ewes where there was an increase in BCS from breeding to embryo recovery (P < 0.07). Similarly, diet had no influence on plasma concentrations of leptin. As anticipated, average concentrations of leptin were greater in heavier than in lighter ewes (P < 0.05). Breeding leptin concentrations were also greater in ewes that increased their BCS (P < 0.01) during the experiment. Neither average plasma insulin nor IGF-I concentrations differed in relation to diet, ewe weight, or BCS. Ewes on the F diet had greater (P < 0.05) plasma NEFA concentrations relative to the controls. Average and breeding NEFA concentrations were also correlated with a positive change in ewe BCS (P < 0.01) and weight (P < 0.05).

Plasma progesterone in *Phase 1 *(mean overall 53.0 ± 1.8 ng ml^-1^) were lower than in *Phase 2 *(mean overall 59.8 ± 1.3 ng ml^-1^). Ewes on the F diet had lower overall progesterone concentrations (P < 0.05) than those fed the C diet in the period between breeding and conceptus recovery (Table 2) as well as in the earliest stages of pregnancy (d2 to d5 period). Paradoxically, the early rise in progesterone concentrations (slope of the curve from d2 to d5 post-estrus), which is a reflection of the onset of CL function, was greater in ewes on the F diet than in those on the C diet (C diet 1.3 ± 0.1 ng ml^-1 ^day^-1^; F diet 1.6 ± 0.1 ng ml^-1 ^day^-1^). Taken together, these results provide evidence that ewes fed the F diet were slower in initiating a progesterone rise, but when they did, the rise was more rapid, although overall progesterone production by their CL was never as great as those ewes fed the C diet.

## Discussion

Numerous types of fat including tallow [39], flaked fat [40], calcium soaps of fat [41], free fatty acids [42] and fish oils [43] have been added to the standard diets of ruminant farm species in order to increase energy intake during lactation and around breeding. Unfortunately, none of the numerous trials conducted to evaluate the value of fat supplementation have reported the sex ratio of offspring born to the animals in the studies. Consequently, it is not clear whether sex ratios were unchanged or whether any skewing was overlooked. Here, we show that a diet enriched in the quantity of rumen-protected PUFA, when provided to ewes for a limited period prior to breeding and over the first two weeks of pregnancy, had a marked ability to bias the normal 1:1 sex ratio of conceptuses toward males on day 13 post-breeding.

The diets (C and F) were isocaloric and were balanced in terms of protein, minerals, and vitamins, with both diets having a similar fatty acid composition. The F diet differed primarily from the C diet in that it contained approximately 2.5 times as much crude fat, largely in the form of PUFAs, the majority of which was protected from metabolism by rumen bacteria and hence absorbable through the ileum [24]. Since at least two-thirds of the fatty acids in the C diet would be hydrogenated by rumen microflora [22,44], the real difference in absorbable PUFA content in the F diet was probably at least five-fold greater than in the C diet. The increased plasma NEFA concentrations in the ewes fed the F diet suggested that the feeding of rumen-protected fat elicited a systemic response. There are other reports consistent with our data in ewes where cattle fed supplemented fat exhibited elevated concentrations of NEFA with generally no change in plasma glucose concentrations [20,45]. Hence the main difference between the two groups of ewes was in the amount of PUFA consumed, suggesting that PUFAs were the component of the diet that that led to the skewing of the sex ratio towards males. An increase in PUFA intake may have also altered the relative proportion of individual fatty acids within the total NEFA concentration measured. In addition, the concentration of plasma volatile fatty acids may have been changed.

The proportion of male conceptuses determined in the ewes on the F diet was significantly (P < 0.001) higher (0.69) compared to the approximate 0.5 value normally encountered in flocks [46] but this figure is comparable to some values noted for fawns born to dominant red deer hinds in good body condition [47,48]. Previously published data showing some skewing towards female lambs in twin pregnancies according to the breeding season [46,49] were not evident in our study. However, our study in ewes ruled out BCS, ewe weight, parity order, time of breeding, and likely dominance as the bases of the sex ratio skewing. Rather, it was the composition of the diet consumed in the peri-conception period that appeared to be responsible for the effect. It seems likely that these results can be extended to wild populations of ruminants where the availability and kind of fats in forage at the time of breeding might be the major factors that influence whether a female conceives a son or daughter. Lack of knowledge of dietary composition during this period may also help to explain the ambiguity surrounding BCS and its positive correlation with an increased number of male offspring [3,48-54].

In theory, skewed sex ratios could result from mechanisms that occur either prior to fertilization, *i.e. *through modulating the ability of either X or Y sperm to reach or penetrate the oocyte, or after fertilization via selective loss of conceptuses of one sex relative to the other [4]. Our experiments were not particularly informative about how increased PUFA intake altered sex ratio. They do show that skewing towards males occurred within the first 13 days of pregnancy, but not precisely when within this period the process was initiated. The biochemical analyses revealed no apparent mechanisms to explain the effect of diet on sex ratio. One favoured explanation for preferential loss of female conceptuses is their increased sensitivity to glucose [2,33,55-58]. However, circulating glucose concentrations in our experiments, although quite variable, were not significantly different between the two diets, although it is still conceivable that local concentrations within the reproductive tract differ according to diet [59,60].

In addition to the lack of strong evidence for a glucose effect, it was clear that male conceptuses did not produce more IFNT than females and did not, therefore, have a post-conception advantage by signaling their presence more robustly to the mother. On the contrary, female conceptuses from ewes on both diets displayed a slight advantage in IFNT production, which agrees with previous reports of sexual dimorphic production of IFNT in early developing bovine embryos [33,58,61]. In addition to conceptus sex, and consistent with other studies [62-64], IFNT production was positively associated with conceptus size. A positive correlation between IFNT production and maternal BCS, irrespective of conceptus sex, was another novel finding of the current study and partially supports the importance of BCS as described by Trivers and Willard [1] but only so far as mothers in better condition, close to optimum, potentially increase their chances of maintaining a pregnancy than ones with a poor BCS. Finally, there were no differences observed in the plasma concentrations of IGF-I, insulin or leptin between ewes on the C and F diets, which would suggest only a subtle effect, if any, of these hormones and their downstream target pathways on sex ratio values. As with glucose, however, we cannot rule out that local rather than plasma concentrations of these hormones favor the development of conceptuses of one sex over the other.

It is possible, that PUFAs, rather than causing changes in metabolic parameters, have a more direct role in governing sex ratio. For example, PUFAs have been proposed to have a positive effect on fertility by causing alterations in follicle growth, ovulation rate, oocyte quality, progesterone synthesis, as well as prostaglandin synthesis and metabolism [20,21,65-69]. One interesting difference between the ewes on the two diets was a lower average circulating progesterone concentration and a delayed, though more rapid, rise in progesterone in ewes fed the F diet. Others have noted a similar delay in progesterone production in dairy cows fed fat-supplements [20,70]. In addition, a PUFA-supplement caused the development of larger dominant follicles in cows and a delay in ovulation relative to the LH surge [66]. One possibility, therefore, is that PUFA supplementation, through its effects on follicular maturation, delays oocyte maturation and the time of ovulation, and these late maturing oocytes, for reasons that are unclear, are more likely to be fertilized by Y-sperm. In a recent study, oocytes that were matured *in vitro *for longer periods than usual and then fertilized, gave rise to more male than female embryos, although the basis of the phenomenon was unclear [71]. Interestingly, delaying breeding relative to the maturation of the dominant follicle may provide more male conceptuses in several species [4], including deer and sheep [71-75] and possibly cattle [76]. Although there was no indication that fertilization was delayed in our experiments, it is intriguing to propose that oocytes from the F group of ewes were advanced in terms of their maturity and had reached a state that they were more likely to be penetrated by Y-bearing sperm.

The increased proportion of linoleic acid (LA 18:2 n-6) in the diet may not only alter the timing of oocyte maturation and ovulation but also the physical properties and signalling ability of the oocyte [69,77]. LA is one of the most abundant fatty acids in ruminant oocytes and embryos [69,78], and is present at two-fold higher concentrations than in human embryos [79]. Recently n-6 PUFAs, which are precursors of eicosanoid signalling molecules, have been shown to play a dominant role in controlling directional sperm motility in the reproductive tract of *Caenorhabditis elegans *[80]. One possibility is that in ewes and related ruminants dietary supplementation of n-6 PUFAs and their accumulation in the oocyte leads to preferential recruitment of Y-bearing spermatozoa.

## Conclusion

In conclusion, the results of the current study suggest that although a greater maternal BCS aids pregnancy establishment it is in fact maternal diet, specifically increased dietary protected PUFA content, which alters sex ratio in favor of males on day 13 post-breeding. Even though the mechanism for sex ratio skewing remains unclear, these data may have considerable practical implications to the livestock industry and to wild life management. Increasing the amount of rumen-protected PUFA and total fat in feed during the breeding period could provide a means of controlling sex ratio of offspring born in the herd or flock. Clearly, however, large-scale breeding trials are needed to determine whether these data can be translated to commercial livestock operations.

## Abbreviations

BCS: body condition score; NEFA: non-esterified fatty acids; LA: Linoleic acid; AA: Arachidonic acid; PUFA: polyunsaturated fatty acids; IGF-I: Insulin-like growth factor I; GnRH: Gonadotropin releasing hormone; LH: Luteinizing Hormone; IFNT: interferon-tau.

## Competing interests

The authors declare that they have no competing interests.

## Authors' contributions

MPG Designed and undertook the animal and *in vitro *studies, cultured and sexed conceptuses, measured the majority of the plasma hormone and metabolites, performed the statistical analysis and wrote the manuscript, LDS assisted with conceptus culturing, sexing and the assaying of plasma hormones and metabolites, TEP helped run the animal feeding and breeding schedule, assessed ewe body condition and the collection of blood samples, KK advised and assisted with conceptus cultures, CNM performed the surgical flushing of conceptuses, JW and MK formulated the diet and feeding regime, JAG aided with the study design, assessed ewe body condition, assisted with the surgical flushing of conceptuses and had input into the manuscript writing, DHK undertook the plasma IGF-I and leptin assays, aided with statistical analysis, the interpretation of data and preparation of the manuscript, RMR conceived the study, secured the funding and participated in its design, as well as the writing of the manuscript. All authors read and approved the final manuscript.
